# The metabolic cost of turning right side up in the Mediterranean spur-thighed tortoise (*Testudo graeca*)

**DOI:** 10.1038/s41598-021-04273-w

**Published:** 2022-01-10

**Authors:** Heather E. Ewart, Peter G. Tickle, William I. Sellers, Markus Lambertz, Dane A. Crossley, Jonathan R. Codd

**Affiliations:** 1 School of Biological Sciences, University of Manchester, Manchester, UK; 2 School of Biomedical Sciences, University of Leeds, Leeds, UK; 3 School of Natural Sciences, University of Manchester, Manchester, UK; 4 Institut Für Zoologie, Rheinische Friedrich-Wilhelms-Universität Bonn, Bonn, Germany; 5 Sektion Herpetologie, Zoologisches Forschungs Museum Alexander Koenig, Bonn, Germany; 6 Department of Biological Sciences, University of North Texas, Denton, USA

**Keywords:** Metabolism, Respiration, Animal physiology, Biomechanics, Herpetology, Ecophysiology, Ecology, Evolution, Physiology

## Abstract

Armoured, rigid bodied animals, such as Testudines, must self-right should they find themselves in an inverted position. The ability to self-right is an essential biomechanical and physiological process that influences survival and ultimately fitness. Traits that enhance righting ability may consequently offer an evolutionary advantage. However, the energetic requirements of self-righting are unknown. Using respirometry and kinematic video analysis, we examined the metabolic cost of self-righting in the terrestrial Mediterranean spur-thighed tortoise and compared this to the metabolic cost of locomotion at a moderate, easily sustainable speed. We found that self-righting is, relatively, metabolically expensive and costs around two times the mass-specific power required to walk. Rapid movements of the limbs and head facilitate successful righting however, combined with the constraints of breathing whilst upside down, contribute a significant metabolic cost. Consequently, in the wild, these animals should favour environments or behaviours where the risk of becoming inverted is reduced.

## Introduction

Testudines are unique among extant amniotes in possessing a bony shell. The evolution of a fully ossified shell was only possible due to a flank-muscle driven ventilatory apparatus that evolved around 50 million-years prior^1^. The shell is formed from the vertebral column, expanded ribs and gastralia that, with the inferred loss of the intercostal muscles^1^, prevents breathing using expansion and contraction of the rib cage. Tortoise inspiration and expiration is predominantly controlled by a ‘muscular sling’ involving two muscles; the *M. obliquus abdominis* and *M. transversus abdominis*^1^. These novel adaptations in abdominal muscle function had to have occurred in the ancestors of turtles long before they evolved their shells^1,2^. Once ventilation no longer required flexibility of the trunk, a constraint on the dorsal ribs was removed, allowing them to further broaden, fuse and eventually form a rigid carapace^1^. Although the method of breathing is strikingly different in turtles they are reported to have a cost of breathing, the energy expended to power the respiratory system, similar to other vertebrates^3^. The cost or work of breathing will impact all aspects of an animal’s daily energy budget including the energetic cost of moving. Breathing in turtles is not correlated to gait cycle during terrestrial locomotion, suggesting a de-coupling of respiration and locomotion^4^.

Shells convey a range of advantages for tortoises including improved protection from predation and environmental stressors^5–7^. However, the trade-offs for having an inflexible vertebral column and rib cage include its influences on tortoise movement and locomotion^1^. Tortoises are famous for their slow locomotion, which is directly related to their rigid and inflexible bodies^8^. Tortoises locomote with diagonally opposite feet moving together so that two feet are in contact with the ground at almost all times^9^. These adjustment in gait are thought to limit body swaying and enable them to avoid fast and energetically expensive movements of the limbs^9^. The metabolic cost of locomotion (CoT) has been directly measured using respiromety in only two Testudines species, *Emydura macquarii* and *Terrapene ornata*^10^. In both these species the CoT was around half of that expected^11^. Tortoise locomotion is notably more energetically efficient than that of other vertebrates of similar mass due in part to their limb muscle physiology^12,13^. The use of only one gait (walking) over their entire limited speed range during locomotion allows the maximum optimisation for energy efficiency of slow muscle fibres^13^.

Aside from the impacts on how they breathe, being limited by their mobility, rigid-bodied and armoured terrestrial Testudines species are also susceptible to overturning through every-day occurrences such as competition, mating behaviour, predation attempts, or locomotion over uneven landscapes^14,15^. Failure to self-right once turned upside-down can lead to life-threatening consequences including predation, starvation, desiccation, loss of mating or foraging opportunities, and thermal stress^14–18^. Within the Testudines there are two strategies for self-righting; (i) using neck extension to rapidly push the body around its longitudinal axis to an upright position^19^, or (ii) when the neck does not extend sufficiently to raise the body above the ground, aggressively shaking the limbs and head to create the momentum to spin the body to an angle at which they can flip to an upright position (Supplementary Movie S1). Research into self-righting has focussed primarily on the influence of body size, body shape and how morphological variations in limb lengths or measures of flexibility habitat structure and ruggedness affect righting ability^15,19–22^. These studies have demonstrated that righting ability is primarily related to carapace structure, shape and size as well as the extension length of the limbs, including the neck^19^. Recent studies have developed theoretical models of the interaction between shell shape and righting^19,23,24^ and the influence of biotic or abiotic factors^25^. Self-righting performance is commonly quantified as the total time taken to right^26^ and is considered an important fitness-related trait^12,27^. Fitness proxies often centre around carapace morphology and modelling of the energetics of righting^27^ and have been used to link self-righting performance to improved survival^28^. However, the actual metabolic cost of self-righting, which is an essential element for assessing fitness, has never been experimentally quantified in any Testudines.

Accordingly, using respirometry and video analysis, we measured the metabolic cost of self-righting (CoR) in female sub-adult Mediterranean spur-thighed tortoise (*Testudo graeca*) that uses movements of the limbs and head to self-right. We placed the CoR into context by comparison to the metabolic cost of transport (CoT) at an average sustainable speed.

## Results

The tortoises we tested were unable to self-right on a hard surface; however, through a set of repetitive movements, the tortoises could effectively turn right side up on a granular substrate (Supplementary Movie S1). Once inverted the tortoises will begin a characteristic set of movements and always self-right via a transversal roll over the longitudinal axis^19^. Rapid waggling (bobbing-lateral movements) of the head, and swinging (lateral movements) of the front and hind limbs begins immediately once inverted. These rapid limb movements, the limbs and head come into contact with the ground which have the effect of turning and rotating the body around in a circular motion, while gouging out a channel in the substrate. As the tortoise creates a channel, often completing a half or full circle, the body is angled to a degree that will allow the limbs closest to the substrate and the head to come into contact with the ground through a series of swinging movements. These movements have the effect of deepening the channel and accompanying mound behind the body against which the tortoise can push the carapace.

It is finally through the use of these swinging movements that the tortoise is able to create a channel deep enough to reach the tipping point of the angle of the carapace against the substrate, that the animal will flip (Supplementary Movie S1).

**Figure 1:**
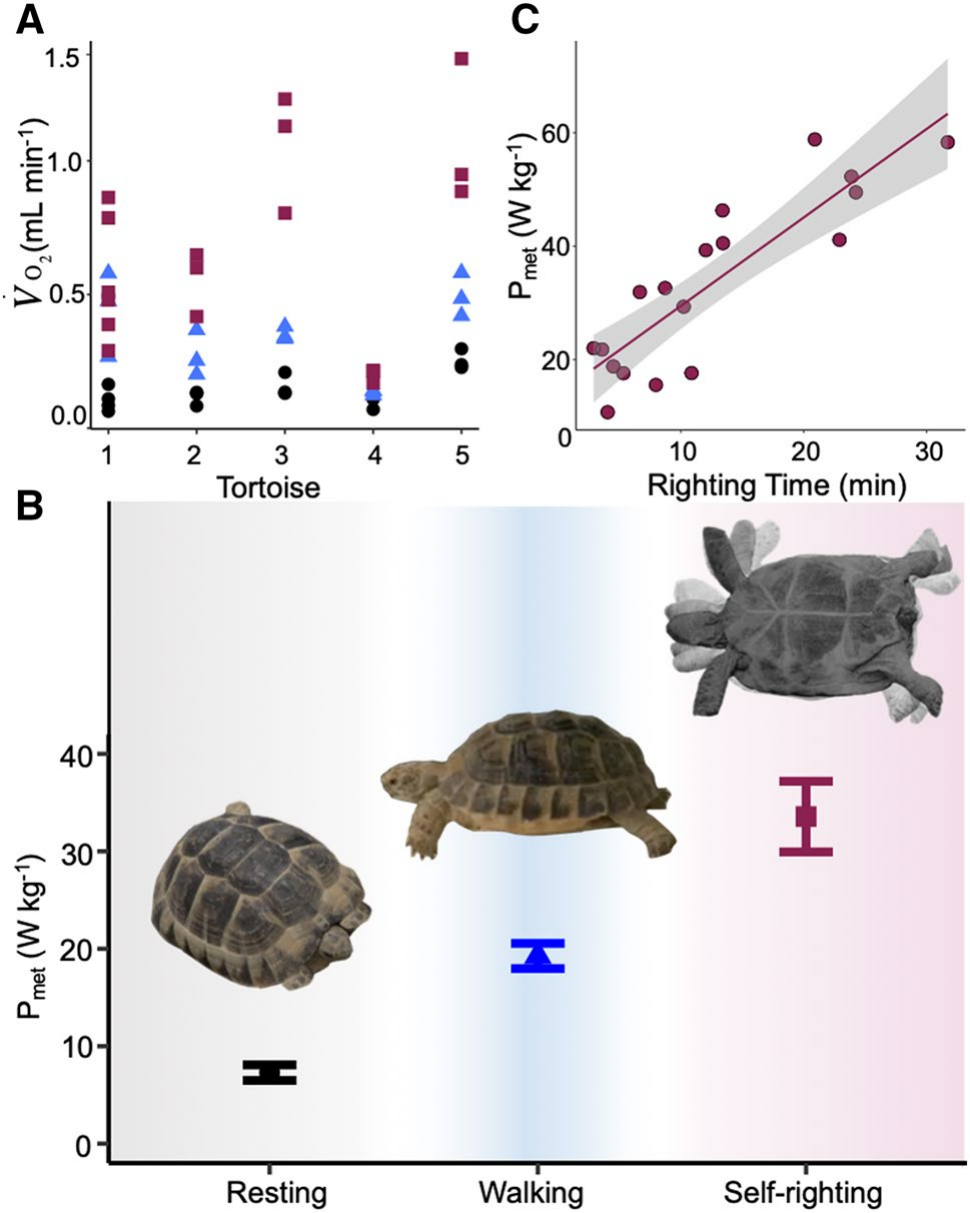
The cost of self-righting and walking (**A**) Rate of oxygen consumption ($\dot{V}{\text{O}}_{2}$, mL min^−1^) and (**B**) Mass specific metabolic power (P_met,_ W kg^−1^) during resting (black circles), walking (blue triangles) and self-righting (maroon squares). Self-righting is the most metabolically expensive behaviour using over two times the oxygen and mass specific metabolic power per kg compared to walking at 6 cm s^−1^ and four to five-fold that at resting. Representative images are snapshots representing the stages correlating to resting, walking and the self-righting process for the Mediterranean spur-thighed tortoise (Supplementary Movie S1). (**C**) Mass-specific metabolic power (P_met_, W kg^−1^) of self-righting tortoises plotted against time (min), demonstrating the linear increase with total time taken to self-right. Shaded region represents the 95% confidence interval. All data mean ± SE. Tortoise images taken by HE.

Respirometry demonstrated that the CoR is relatively energetically expensive ($\dot{V}{\text{O}}_{2}$ 0.66 ± 0.09 mL min^−1^, P_met_ 33.59 ± 3.61 W kg^−1^), at over 2 times CoT ($\dot{V}{\text{O}}_{2}$ 0.34 ± 0.04 mL min^−1^, P_met_ 19.71 ± 1.26 W kg^−1^) and four to five-fold greater than resting metabolic rate (RMR) ($\dot{V}{\text{O}}_{2}$ 0.15 ± 0.02 ml min^−1^, P_met_ 8.27 ± 0.91 W kg^−1^) (Fig. 1a,b, Supplementary Tables S1, S2). Interestingly, kinematic analyses showed maximum limb movement frequency during righting (13.99 ± 4.10 Hz, Supplementary Movie S1) was five-fold higher than that during walking at a speed of 6 cm s^−1^ (2.72 ± 0.31 Hz, Supplementary Movie S2). In addition, there was also a link between total righting duration time (12.6 ± 2.02 min) and the power requirement, with the P_met_ increasing linearly with the time taken to right (Fig. 1c). All data mean ± SE.

## Discussion

**Figure 2:**
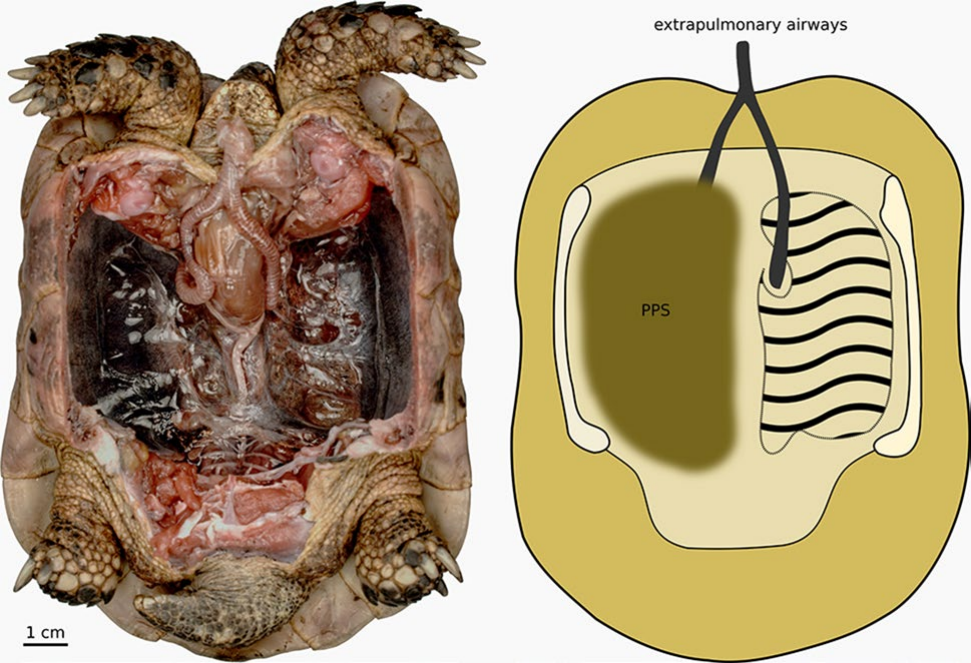
The Post Pulmonary Septum in *Testudo* Overview of the coelomic subdivision and the resulting compartmentalization of the respiratory system in tortoises. The left image shows in a ventral view the viscera, except for the lungs, have been removed. The schematic drawing on the right indicates the structures in question. Unlike in most other clades of chelonians, the ventral side of the lungs is completely covered by the postpulmonary septum (PPS). The anatomically left side shows the medial parts of the PPS removed, giving a clear view on the actual lungs beneath (i.e. dorsal to) it. Dorsally, the lungs are broadly fused to the inner side of the carapace (indicated by the wavy lines), resulting in a firm fixation within the pleural cavity. Note the PPS extending laterad to the carapace. The anatomical configuration serves as a mechanical support system in inverted tortoises whose viscera are pushing on the lungs. As a consequence, the PPS prevents gravitational pulmonary collapse in inverted specimens and hence counteracts the resulting decrease in compliance together with the associated increase in work of breathing. Photograph taken by ML.

Self-righting is an intriguing behaviour in Testudines and one that is important for understanding such fundamental aspects of their biology as the evolution of their shell shape^12,19^, survivability of hatchlings^28^. The shells of Testudines are an evolutionary novelty. The rigid shell is a relatively fixed volume, which means that anything that alters the space available inside the shell could restrict lung volume and ultimately influence breathing. Despite having the pelvic and pectoral girdles inside the shell, through their specialised abdominal ventilatory mechanism, terrestrial Testudines locomotion performance does not appear to conflict with breathing performance^29^. However, our data indicate that self-righting incurs a significant energetic expense, relative to walking, that suggests there may be an as yet unconsidered novel constraints associated with having evolved a shell. During inspiration the action of the oblique muscle is aided indirectly by a gravity-induced ventral pull on the liver, that facilitates inflation of the lungs^30^. However, being upside down reverses the direction of gravity acting on the liver. Coupled with the increased mass of the viscera, predominantly the stomach that along with the liver is directly connected via inter-organ connections to the ventral side of the lungs^31^, being inverted likely constricts the ability of the lung to inflate thereby reducing lung volume or at the very least decreases pulmonary compliance. Submersion in aquatic species has an analogous effect in reducing lung volume and ultimately increases the work of breathing^32^. Any increases in the work of breathing would contribute to the metabolic cost of self-righting. Although being inverted would act to reduce lung volume, the post pulmonary septum (PPS, Fig. 2) likely prevents total collapse of the lung. Interestingly the PPS is reduced in species such as the common snapping turtle (*Chelydra serpentina*)^31^ that use rapid neck driven self-righting^5^, suggesting future research into any functional linkage between degree of lung coverage by the PPS and the adoption of a self-righting strategy would be beneficial.

The energetic cost of behaviours relevant for free-ranging animals is often overlooked, including how these might vary during ontogeny, however, these studies provide a crucial link between physiology and ecology^33^. The cost of movement can be a substantial and ultimately any energy used for movement isn’t available for other functions, creating a trade-off against the energy available for growth, feeding, thermoregulation and/or reproduction^33,34^. Therefore, each of these components must be serviced while attempting to ensure that overall energy output ≤ energy input. Energy input is not infinite and metabolic scope (the difference between resting and maximal metabolic rates) is limited under real conditions^34^. Given the option animals will tend to make decisions that minimise their energy output for a given task^35^ and will make appropriate choices on how and where to move within an energetic landscape^36^ suggesting that for tortoises they should seek to minimise the energy being expended by avoiding inverting. Generally, there are very few studies examining the energetics of any behaviours in Testudines. The metabolic cost of transport, for example, has only been examined in two species (prior to the single speed reported in the current study) in a terrestrial (Cryptodira: *Terrapene ornata*^10^) and aquatic (Pleurodira: *Emdura macquarii*^37^) species. Both these studies demonstrated that metabolic cost of locomotion was surprisingly lower than expected for their body mass and when compared to other amniotes^10,37^. Our data support the previous findings that testudines are able to move very efficiently.

Locomotion is a significant component of the daily activity and energy budgets of most animals, including tortoises^38^. The CoT has been studied across a wide range of animals and is primarily determined by the energetic cost of generating muscle force to support and propel body weight^39^. CoT is optimised by increasing the metabolic efficiency of muscle contractions and by minimising the mechanical work done during locomotion^40,41^. The unique anatomy of the Testudines has eliminated their need for the muscular sling found in other quadrupeds^42^, which may serve as an energy saving mechanism during their movement^10^. Slow moving tortoise terrestrial locomotion is more efficient than that of other vertebrates of a similar mass, due in part to their muscle physiology^11,13,43^. The predominance of type I fibres in tortoise locomotor muscles, that are known to stretch and contract at a slower rate than other vertebrates^13^, is adaptive for lower intensity movements such as walking. Slow contracting tortoise muscles are in fact the most efficient ever studied, with 35% of available chemical energy converted to mechanical work during contraction^13^; this compares to thermodynamic efficiency of only c.20% in mouse and dogfish muscle^44^. The energetic efficiency of muscle is inversely related to shortening speed^45^, with force generation notably more efficient in slow twitch type I muscle fibres compared to fast twitch type II^45^. The evolution of the shell has allowed tortoise to avoid having to increase locomotor speed to escape; however these adaptations for moving slowly and efficiently do not appear compatible with minimising the metabolic cost of self-righting. The rate at which muscles use metabolic energy increases sharply with increasing rate of contraction. Therefore, the rapid neck and limb movements during self-righting (Supplementary Movie S1), come at the cost of approximately doubling the P_met_ (relative to walking), which can be explained by the increase in oxygen consumption with increases in muscle contraction rate and the recruitment of faster twitch fibres^45^. The linear increase in P_met_ with the time taken to right (Fig. 1c) is an interesting phenomenon. It suggests that the power employed, in other words the effort that the animal makes, increases as the time the animal has spent inverted increases. This observation supports previous estimations using time as a proxy for energetic cost^5^.

## Conclusion

Overcoming the dangers of inverting is a unique challenge faced by tortoises as armoured-bodied tetrapods. Our data indicate that self-righting requires a specific set of biomechanical movements to be successful and is a relatively metabolically costly behaviour in sub-adult female *T. graeca.* Sexual dimorphism in righting performance, with males righting faster and more often than females on average, has been documented in tortoises^17^; and it would be interesting to determine in future work if similar sex differences were found in the metabolic cost of self-righting. In sub-adult female *T. graeca* in our study the relatively high cost can be explained by the constraints of breathing upside down and the increased muscular work required for rapid limbs movements during self-righting. Research is needed on the frequency or risk of becoming inverted for any individual Testudines species. However, our data strengthen the links made between the fitness proxies associated with righting ability, and may ultimately allow us to better understand route choice and movement patterns. Our data would predict that in the wild, tortoises should eschew environments and behaviours where they are more prone to inverting to avoid the increased metabolic costs of turning right side up.

## Methods

### Animals

All experimental protocols were performed in accordance with the relevant guidelines and regulations and approved by the University of Manchester Research Governance, Ethics and Integrity Committee (Permit D.039). Animal care and experimentation were also in accordance with the ARRIVE guidelines. Captive born, 2-year-old female Mediterranean spur-thighed tortoises (*Testudo graeca, n* = *5*) were housed in the Biological Services Facility at the University of Manchester. Tortoises were maintained indoors in a vivarium (2 × 0.9 × 0.7 m) under UV and natural light bulbs, and ceramic heating lamps set up to provide a thermal gradient from hot (35 °C) to cool (25 °C) regions^46^. Body mass was recorded at the start of trials (Electronic Supplementary Material, ESM). Fine-grain sand was selected as a substrate representative of natural conditions and tortoises were provided with rocks, wooden logs and tunnels for enrichment. Tortoises were fed a mix of commercial tortoise pellets (Komodo Complete Holistic Tortoise Diet, Happy Pets Products, Leicester, UK) with mixed fresh vegetables and dark leafy greens. Cuttlefish bones were also provided. Because tortoise digestion can take up to several weeks to months^47^, individuals were not fasted prior to experimentation. Food and water were available ad libitum.

### Experimental protocol

Ectothermic physiological performance is temperature-dependent^48^, and optimal performance occurs around 35 °C in reptiles^49^. Therefore, tortoises were maintained in the tank hot zone immediately prior to all experimentation and body temperature averaged 34.6 ± 0.4 °C during all trials. The same tortoises were then examined under two separate experimental protocols to measure the CoR and the CoT. For the CoR trials a randomly selected tortoise was placed into the righting chamber (40 × 30 × 20 cm) filled with fine sand to a depth of 10 cm (Supplementary Movie S1). Once gas concentrations became stable during the resting period we measured a resting metabolic rate (RMR), then the chamber lid was then quickly lifted, the tortoise placed into an inverted position and the lid replaced. After self-righting the individual was then left in the chamber to settle and record a second RMR before removal. For the CoT trials, tortoises were trained for 4 months to walk inside a Perspex^®^ box (20 × 15 × 10 cm) on a small animal treadmill (Model: LE8710, Panlab, Harvard Apparatus, Spain). Preliminary trails indicated that the tortoises were restricted to a walking gait over a limited speed range (3–10 cm s^−1^). When freely moving along a race during initial training the tortoises preferentially chose to move at mid-range speeds of 5–7 cm s^−1^. Therefore, we selected a mid-range, easily sustainable speed (6 cm s^−1^, Supplementary Movie S2) for comparison to CoR. During walking trials, a randomly selected tortoise was placed onto the treadmill to record RMR, then walked at 6 cm s^−1^ until a steady trace was achieved (3–5 min) after which time a second RMR trace was recorded. Replicates for each tortoise were recorded for CoR and CoT of between 2 and 6 trials, and tortoises were given a rest day in between trials. For both CoR and CoT experiments, the lowest value was defined as RMR.

### Respirometry and kinematics

Oxygen consumption ($\dot{V}{\text{O}}_{2}$) and carbon dioxide production ($\dot{V}{\text{CO}}_{2}$) were measured using an open-flow respirometry system (all equipment and software: Sable Systems International^®^, Las Vegas, NV, USA). Air was pulled through the respirometry chamber at 2 L min^−1^ and 0.3 L min^−1^ (CoR and CoT, respectively) using a mass flow pump (MFS-2). Water vapour pressure of excurrent air was then measured (RH300 water vapour meter) before water was removed from the airstream using calcium chloride (2–6 mm granular, Merck, Germany) after which CO_2_ content was measured (CA-10a CO_2_ analyser). Soda lime (2–5 mm granular, Sigma-Aldrich, Germany) was then used to scrub CO_2_ and finally O_2_ was measured (Oxzilla II absolute and differential dual channel O_2_ analyser). Each system was calibrated using a known flow rate of nitrogen, and found to be accurate to between ± 3–5% for both experimental set-ups. The primary flow rate was adjusted to a dry-corrected flow rate and oxygen consumption and carbon dioxide production calculated using the appropriate equations^50^. $\dot{V}{\text{CO}}_{2}$ was then used with $\dot{V}{\text{O}}_{2}$ to calculate the respiratory exchange ratio (RER = $\dot{V}{\text{CO}}_{2}$:$\dot{V}{\text{O}}_{2}$). $\dot{V}{\text{O}}_{2}$ was then converted to mass-specific power consumption (P_met_, W kg^−1^) by multiplying $\dot{V}{\text{O}}_{2}$ by the calorific RER equivalent (in Joules) taken from (Table 12.1^51^). Data were processed using ExpeData^®^ using a 60-s segment of a steady state trace during CoT and CoR trials. For analysis of total mass specific power over the CoR trial the entire steady state section of the trace was used. A Sony cyber-shot camera (DSC-RX-10 III, Sony Corporation^®^, Tokyo, Japan) was used to film all trials at 100 fps during self-righting and walking. Tracker v.2.51 (https://physlets.org/tracker; The Open Source Physics Project) was used to analyse limb movement frequency (MF; Hz).

### Data analysis

All statistical analyses (Supplementary Table S2) were performed using R statistical software (version 3.6.2, R Core Team 2020, http://www.R-project.org/). Differences in $\dot{V}{\text{O}}_{2}$ and P_met_ between resting, walking, and righting tortoises, and frequency of limb movements between walking and righting tortoises, were examined using a one-way ANOVA with a post-hoc Tukey HSD test. The relationship between P_met_ and time (min), was investigated using a linear regression. Statistical differences were considered significant when *p* < 0.05.

## Supplementary Information

Supplementary Information 1.

Supplementary Video 1.

Supplementary Video 2.

Supplementary Information 2.
