# Supplementary material for: The metabolic cost of turning right side up in the Mediterranean spur-thighed tortoise (Testudo graeca)
Source: Sci Rep. 2022 Jan 10;12:431. doi: 10.1038/s41598-021-04273-w (PMC8748805; doi:10.1038/s41598-021-04273-w)
Supplement: Supplementary file 1 — Supplementary Information 1. [file 41598_2021_4273_MOESM1_ESM.docx]

**Electronic Supplementary Material Legends**

**Movie S1 –** **Mediterranean spur-thighed tortoise, *Testudo graeca,* self-righting**. Once inverted the tortoise begin rapid limb and head movements which will move the body in a circular motion until they gouge a channel out facilitating the self-righting. The plastic righting chamber used for respirometry has been removed to enable the tortoise behaviour to be clearly seen.

**Movie S2 – Mediterranean spur-thighed tortoise, *Testudo graeca,*** walking at 6cm s^-1^. Tortoises were trained for four months to walk at a steady pace. The Perspex® box used for respirometry has been removed for to enable the tortoise behaviour to be clearly seen.

**Table S1** – **Raw and processed respirometry and kinematics dataset** for *Testudo graeca* during self-righting and walking at a moderate sustainable speed. See excel spreadsheet: Ewart et al_Raw and processed respirometry and kinematics dataset.xlsx.

**Table S2** – **Statistical output** – significant differences were found between mean oxygen consumption (mL min^-1^) and mean mass-specific power consumption (P_met_ W kg^-1^) between resting, walking, and self-righting tortoises. and P_met_ were highest in self-righting tortoises.

| Analysis | Variables | Mean ± SE | Statistic & p-value |
| --- | --- | --- | --- |
| One-Way ANOVA | Resting | 0.15 ± 0.02 | F=28.6, df=2,45 p<0.001 |
|  | Walking | 0.34 ± 0.04 |  |
|  | Self-Righting | 0.66 ± 0.09 |  |
|  |  |  |  |
| One-Way ANOVA | Resting P_met_ | 8.27 ± 0.91 | F=49.1, df=2,45, p<0.001 |
|  | Walking P_met_ | 19.71 ± 1.26 |  |
|  | Self-righting P_met_ | 33.59 ± 3.61 |  |
|  |  |  |  |
| Linear Regression | P_met_, Time |  | Adj. R-squared=0.743, df=1,16, p<0.001 |
|  |  |  |  |
